# Controlled Signaling—Insulin-Like Growth Factor Receptor Endocytosis and Presence at Intracellular Compartments

**DOI:** 10.3389/fendo.2020.620013

**Published:** 2021-01-29

**Authors:** Leonie Rieger, Rosemary O’Connor

**Affiliations:** School of Biochemistry and Cell Biology, BioScience Institute, University College Cork, Cork, Ireland

**Keywords:** insulin-like growth factor 1 receptor (IGF-1R), signaling, endosomes, nucleus, Golgi

## Abstract

Ligand-induced activation of the IGF-1 receptor triggers plasma-membrane-derived signal transduction but also triggers receptor endocytosis, which was previously thought to limit signaling. However, it is becoming ever more clear that IGF-1R endocytosis and trafficking to specific subcellular locations can define specific signaling responses that are important for key biological processes in normal cells and cancer cells. In different cell types, specific cell adhesion receptors and associated proteins can regulate IGF-1R endocytosis and trafficking. Once internalized, the IGF-1R may be recycled, degraded or translocated to the intracellular membrane compartments of the Golgi apparatus or the nucleus. The IGF-1R is present in the Golgi apparatus of migratory cancer cells where its signaling contributes to aggressive cancer behaviors including cell migration. The IGF-1R is also found in the nucleus of certain cancer cells where it can regulate gene expression. Nuclear IGF-1R is associated with poor clinical outcomes. IGF-1R signaling has also been shown to support mitochondrial biogenesis and function, and IGF-1R inhibition causes mitochondrial dysfunction. How IGF-1R intracellular trafficking and compartmentalized signaling is controlled is still unknown. This is an important area for further study, particularly in cancer.

## Introduction

**Figure 1:**
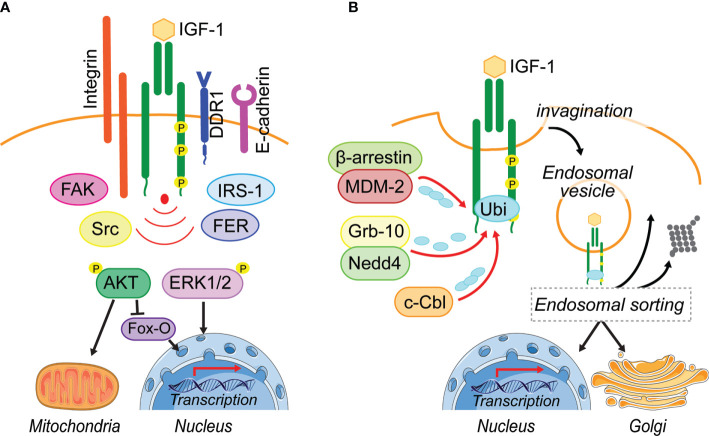
Leaving the plasma membrane. **(A)** Located on the plasma membrane, activated IGF-1R induces two major pathways, PI3-K/AKT and MAPK/ERK1/2, to regulate cellular processes including metabolism and transcription. Different adhesion related kinases (FAK, Src, FER) and interacting proteins (IRS-1, DDR1) regulate IGF-1R endocytosis and thereby prolong or reduce IGF-1R signaling from the cell surface. In addition, these IGF-1R interacting proteins can enhance bias IGF-1R signaling or their cooperation is needed for the activation of IGF-1-induced pathways (Integrin). **(B)** Ligand-induced IGF-1R activation leads to the recruitment of E3-liages (MDM-2, Nedd4, c-Cbl) that can initiate IGF-1R poly- and mono-ubiquitination. Via membrane invagination and formation of clathrin- and caveolin-coated pits, the IGF-1R enters the cell in endosomal vesicles. It is assumed that the endosomal sorting system decides, whether IGF-1R gets degraded, travels back to the plasma membrane or translocates to intracellular membrane compartments. To this day it is unknown how the post-endocytotic IGF-1R translocation to intracellular membrane compartments, such as the Golgi and the nucleus is regulated and whether IGF-1R regulation of mitochondrial function is exclusively due to signaling transduction. Figure elements adapted from Servier Medical Art (https://smart.servier.com/), under license CC-BY3.0.

Insulin-like growth factor-1 (IGF-1) stimulates essential cellular processes including proliferation, differentiation, survival and metabolism and thereby is essential for normal growth and development. Upon IGF-1 binding to the IGF-1 receptor (IGF-1R), the kinase domain becomes activated, leading to autophosphorylation of specific tyrosine residues (1–4). The subsequent recruitment and phosphorylation of Insulin-receptor-substrate (IRS-1 and IRS-2) proteins (5, 6) facilitates recruitment of PI3-Kinase and activation of the AKT-mTOR pathway (**Figure 1A**). This conserved signaling pathway regulates metabolism and transcription to promote cell survival growth or proliferation (7, 8). Activated IGF-1R may also recruit Src homology and Collagen (SHC) adaptor proteins (6, 9), and IGF-1-induced SHC phosphorylation leads to activation of RAS and the MAPK pathways that mediate mitogenic, differentiation, and migratory signals (10, 11).

IGF-1R activity can facilitate tumorigenesis, maintenance of the transformed phenotype and cancer progression (12, 13). Furthermore, IGF-1 may stimulate cancer cell migration, acquisition of epithelial-mesenchymal transformation (EMT) and chemotherapy resistance. Unsurprisingly, targeting the IGF-1R has been extensively investigated as a strategy in cancer therapy. Several kinase inhibitors and blocking monoclonal antibodies that inhibit ligand binding and signal transduction, while also triggering downregulation of the receptor have been tested (14, 15). However, the fact that these inhibitors have been largely unsuccessful in clinical trials renewed attention on how regulation of IGF-1R internalization, subcellular location and signaling are controlled in normal and cancer cells.

Although once thought that when cell surface RTKs are internalized, their signal transduction is terminated, it is now generally accepted that internalized receptors, including the IGF-1R may signal from endosomal and intracellular membrane compartments, or may also regulate gene transcription by translocating to the nucleus (16–22). However, the mechanisms of intracellular trafficking and which signals determine the subcellular localization of the IGF-1R or its compartmentalization with other signaling proteins are not known. Recent studies suggest that these events are regulated in a cell type-specific way and that cell-specific signals may influence the recruitment and activation of effector proteins (20, 22). Therefore, the cell-specific IGF-1R trafficking, compartmentalization and its subcellular location may define how cells respond to different extracellular stimuli.

Here, we review recent work on IGF-1R endocytosis, post-endocytotic trafficking and IGF-1R signaling to and from intracellular membrane compartments. We review how a non-canonical trafficking pathway *via* translocation of the receptor to internal membrane compartments and its signaling from the Golgi apparatus may contribute to its activity in cancer cells. Finally, we review the functions of IGF-1R presence in the nucleus and its effects of IGF1 signaling on mitochondrial activity.

## Leaving the Plasma Membrane-Insulin-Like Growth Factor 1 Receptor Ubiquitination and Endocytosis

Whether the IGF-1R undergoes ligand-induced endocytosis or remains on the plasma membrane is determined by the recruitment of interacting proteins (**Figure 1A**). It has been suggested that under pathological conditions like cancer, the IGF-1R associates with a range of other receptor and signaling complexes at the plasma membrane (23, 24). In particular, adhesion receptors and kinases, known to associate with the IGF-1R include E-cadherin (25), β1-Integrin (26), the discoidin domain receptor 1 (DDR1) (27), focal adhesion kinase (FAK) (28, 29), Src (30), the feline-sacroma-related kinase (FER) (31). All of these have been implicated in modulating IGF-1R stability or endocytosis to promote specific cellular responses (**Figure 1A**). However, it is unknown whether or how they might influence IGF-1R endosomal trafficking.

As with other RTKs, IGF-1R endocytosis is initiated by vesicle formation on the membrane (**Figure 1B**), and endocytosis *via* clathrin-coated-pits (CCP) is considered to be the fastest and predominant mode of internalization (23, 24, 32). The formation of CCPs requires recruitment of proteins that contain a ubiquitin-interacting motif, such as epsin, Eps15, or AP-2, to the activated receptor (23, 24, 32). Once clathrin-dependent endocytosis is saturated due to a large number of surface receptors being activated, it has been proposed that alternative endocytosis mechanisms subsequently facilitate IGF-1R internalization (33–35).

A clathrin-independent mechanism of endocytosis has been described for ligand-activated EGFR *via* micro- and macropinocytic vesicles. This involves the reorganization of the cytoskeleton and dynamic membrane ruffling (36–38). Although a similar process could be possible for IGF 1R endocytosis, it has not been demonstrated. However, clathrin independent IGF-1R endocytosis also involves the formation of lipid rafts/caveolae, which are generally described as plasma membrane invaginations. Indeed, IGF-1R has been shown to co-localize with the phosphorylated version of caveolin-1, the main component of these lipid rafts (35, 39).

Ubiquitination of the β-subunit of the IGF-1R is associated with initiation of IGF-1R endocytosis (24, 35, 40). This is dependent on IGF-1R kinase activity and requires the presence of the receptor C-terminal tail (35, 41).

Four E3 ligases have been described to either directly or indirectly interact with IGF-1R to facilitate its ubiquitination. The least studied in the context of IGF-1R is HRD1, which functions in the endoplasmic reticulum (42, 43), whereas the others, Nedd4 (40, 44), MDM2 (35, 45–47) and c-Cbl (39), are well studied (**Figure 1B**). IGF-1R ubiquitination can be observed within the first 5 min of ligand-binding. Two IGF-1R ubiquitination sites at Lys^1138^ and Lys^1141^ located within the kinase domain are believed to be the key lysine residues for ubiquitination (48). It is proposed that MDM2 recruitment to the IGF-1R occurs when low amounts of IGF-1are available, leading to IGF-1R endocytosis *via* clathrin, while high IGF-1 concentrations may initiate c-Cbl-mediated ubiquitination of the receptor followed by endocytosis using the caveolin/lipid raft route (39). This supports the idea that alternative endocytosis mechanisms are activated to internalize the IGF-1R, once clathrin-dependent endocytosis is saturated (33–35). A protein complex consisting of MDM2 and the β-arrestin protein links K63-conjugated ubiquitin polypeptide chains to the IGF-1R. This mode of ubiquitination is generally associated with cell signaling responses, DNA repair and protein trafficking (49–51) (**Figure 1B**). c-Cbl attaches K48-conjugated ubiquitin polypeptide chains to the IGF-1R, which may initiate degradation of the receptor (51) (**Figure 1B**). Thus, it is possible that depending on available IGF-1 levels, different E3 ligases are recruited to the receptor to initiate ubiquitination.

Although IGF-1R kinase activity is clearly essential for recruiting the proteins that facilitate receptor internalization and ubiquitination, it is not understood how the C-terminal tail contributes to ubiquitin-mediated IGF-1R trafficking and degradation. Our recent study showed that IGF-1-promoted phosphorylation of the Tyr^1250/1251^ site in the IGF-1R C-terminal results in enhanced IGF-1R internalization and proteosomal degradation (22). However, whether the Tyr^1250/1251^ phospho-site is involved in or modulates IGF-1R ubiquitination is still unknown. The C-terminal tail contains three lysines that are putative sites for ubiquitination, but this has not been demonstrated in cells. It remains possible that phosphorylated Tyr^1250/1251^ could provide a binding site for adaptor proteins or an E3 ligase that targets these sites. This would implicate the activity of domains of the receptor other than the kinase in regulating IGF-1R internalization and trafficking.

### Travel Direction-Determining Insulin-Like Growth Factor 1 Receptor Trafficking Routes

CCP/caveolin-vesicles that contain internalized IGF-1R become fused with early endosomes (27, 40, 44, 52). Here the IGF-1R proteins are sorted, either targeted for degradation (24, 35), transported toward the Golgi network (22), transported to the nucleus (20, 53–56), or recycled back to the plasma membrane (57) (**Figure 1B**). Internalized ubiquitinated proteins can be detected by distinct multiprotein complexes that comprise the endosomal sorting complex required for transport (ESCRT) (58–61) and serve as signal for cargo sorting (58). The fate of internalized proteins to either undergo degradation or recycling is determined within the endosomal sorting network (61). Before membrane cargo within the early endosomes, is submitted to several rounds of cargo sorting, as the early endosome matures into a late endosome (62), cargo destined for the fast recycling route is sorted and delivered back to the cell surface (63). There is also a slow recycling route where proteins first traffic through the recycling compartments before moving back to the cell surface.

Emerging evidence indicates that cargo may also enter a retrograde trafficking route where it is transported back to the Golgi apparatus, a process that serves to maintain a robust membrane protein delivery along the Golgi-associated microtubules (18, 64–66). This particular transport route is important for β1-Integrin-promoted cell migration and adhesion (65). Although precise details of IGF-1R sorting mechanisms and which proteins are involved is still unknown, it is clear that the endosomal network is essential for selecting internalized IGF-1R and its trafficking to distinct cellular compartments. The IGF-1R also travels on a path to the Golgi apparatus as a response to IGF-1-induced phosphorylation at Tyr^1250/1251^ (22). This enhances the potential for distinct intracellular signaling responses from the IGF-1R in different cells and different physiological or pathological settings.

## Back to the Start—The Golgi Apparatus as a New Insulin-Like Growth Factor 1 Receptor Signaling Compartment

**Figure 2:**
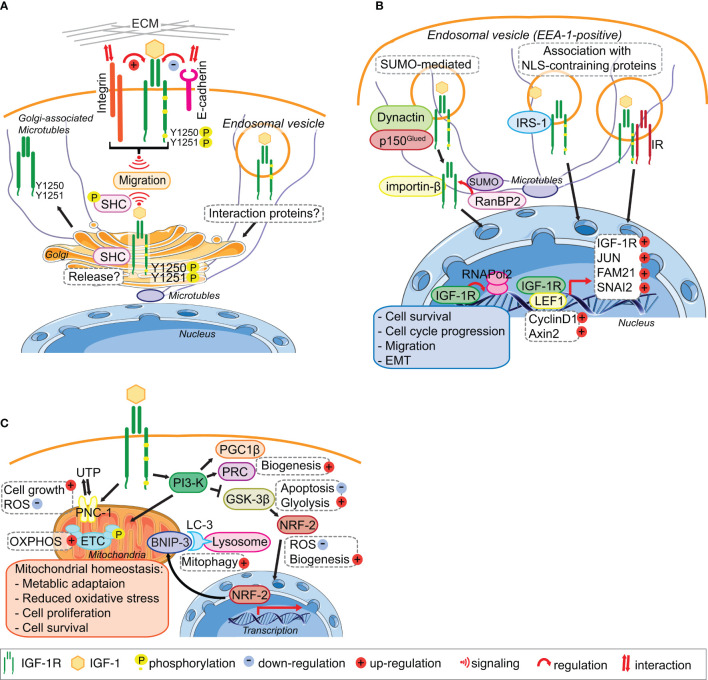
IGF-1R trafficking routes and signaling to the mitochondria and from the Golgi and the Nucleus. **(A)** The IGF-1R translocates to the Golgi apparatus. In migratory cell lines, IGF-1R autophosphorylates Tyr^1250/1251^ in an adhesion dependent manner. Phosphorylation of Tyr^1250/1251^ IGF-1R leads to rapid IGF-1R endocytosis leads to activation of the MAPK pathway and results in translocation of the IGF-1R to the Golgi which promotes sustained SHC activation to facilitate migration. points. The release and retention of IGF-1R in the Golgi may be regulated by β1-Integrin and its interaction with the ECM. In cells with low or no migratory capacity, IGF-1R remains on the surface inducing signaling from the membrane. The interaction with other proteins, including E-cadherin, stabilizes the adhesion points and internalization rate of the IGF-1R is low. **(B)** IGF-1R translocates to the nucleus. IGF-1 binding to the IGF-1R induces the translocation of the membrane receptor to the nucleus. Various mechanisms have been proposed for the import of the IGF-1R to the nucleus. Nuclear IGF-1R can bind to DNA and enhance or initiate the transcription of various genes, leading to cell survival, migration, EMT and cell cycle progression. **(C)** IGF-1 signaling regulates mitochondrial function. The activation of the PI3-K pathway in response to IGF-1 induces the expression of the mitophagy regulators PGC1β and PRC. Inhibition of GSK-3 β by PI3-K activation leads to the release of NFE2L2/Nrf2, which translocates to the nucleus to enhance the expression of the mitophagy receptor BNIP-3. Activation of IGF1-R also enhances the expression of the UTP importer PNC-1, which was linked to cell growth and the reduction of ROS. Through these pathways IGF-1 signaling contributes to the maintenance of mitochondrial homeostasis. Figure elements adapted from Servier Medical Art (https://smart.servier.com/), under license CC-BY3.0.

The Golgi apparatus has a long-understood function in distribution, modification and secretion of newly synthesized proteins. However, it is also intimately involved in cellular processes such as cell polarization (67), directional migration (68), stress (69) and DNA repair (70). Cell migration requires coordinated communication between the plasma membrane and the Golgi apparatus (68). This may be facilitated by the retrograde trafficking of internalized plasma membrane proteins back to the Golgi apparatus (65, 71). This retrograde trafficking enables persistent cell migration because Golgi-derived microtubules act as a fast-track lane to deliver essential proteins to cell migration hot-spots, such as the sites of focal cell adhesion (**Figure 2A**) (64–66). Several key signaling proteins including Ras/MAPK (72–74) and RTKs, including MET, KIT, VGFR2, EGFR, FGFR (21, 75–77) and IGF-1R (22) have been demonstrated to locate to the Golgi apparatus, which acts as a signaling hub in normal and cancer cells (**Figure 2A**).

The rapid endocytosis and subsequent translocation of the IGF-1R to the Golgi in fibroblasts and cancer cell lines requires an adhesion-dependent autophosphorylation on Tyr^1250/1251^ in the C-terminal tail (**Figure 2A**). Although evident in all cells tested, Golgi-localized IGF-1R is however a particular feature of migratory cancer cells, because cancer cell lines with low or no migratory capacity exhibit little less Golgi-localized IGF-1R. Golgi-derived IGF-1R signaling might therefore contribute to aggressive cancer cell behavior (22). In migratory cancer cell lines, IGF-1-induced SHC phosphorylation, which is required for cell migration, is dependent on an intact Golgi apparatus and also requires cell contact with the extra-cellular matrix (ECM), suggesting that the IGF-1R mediates communication between the plasma membrane and Golgi. IGF-1-induced cell migration also requires an intact Golgi apparatus (22), as well as cooperative signaling between the IGF-1R and β1-Integrin (26, 78–81) (**Figure 2A**). β1-Integrin connects the ECM with the actin cytoskeleton of cells and thereby has both a structural and signaling function in cell adhesion and migration (82, 83). This suggests, that in migrating normal and cancer cell lines β1-Integrin signaling from the plasma membrane can influence IGF-1R distribution within cells and determine its presence at the Golgi apparatus (**Figure 2A**).

While β1-Integrin is a strong candidate for determining IGF-1R translocation to and its release from the Golgi in migratory cells (**Figure 2A**), E-cadherin is a strong candidate for enhancing IGF-1R stability and plasma membrane location in low- or non-migratory cell lines. E-cadherin, which is often repressed in migratory cancer cell lines and upon EMT, especially in triple negative breast cancer cells, is readily detectable in a complex with the IGF-1R at sites of cell–cell contact in cancer cells with no or low migratory capacity (25, 84). However, in confluent migratory cancer cells (with evident high levels of cell-cell contact), and under conditions where cells are unable to migrate, the IGF-1R remains in the Golgi apparatus. Therefore, E-cadherin expression in cancer cells with no or low migratory capacity may limit IGF-1R translocation to the Golgi apparatus. Regulated and exclusive expression of cadherins and Integrins has been linked to the migratory capacity of cells during embryonic development, tumor invasion and metastasis (85–87).

Thus, it is likely that IGF-1R function in facilitating cell migration through its translocation to and signaling from the Golgi is influenced by adhesion related proteins that are expressed differently depending on cell type, which may be influenced by their hormone receptor expression, and fate, as it has already been proposed (23, 24). However, the mechanisms of this interplay between adhesion receptors and IGF-1R trafficking to and from the Golgi are still unknown. It is not known how phosphorylation and or dephosphorylation of key residues on the receptor control this and how the array of signaling proteins present at the Golgi interact.

## Journey to the Center of the Cell- Insulin-Like Growth Factor 1 Receptor in the Nucleus

Several RTKs have been observed in the nucleus of cancer cells. These include EGFR family members (88–90), FGFR1 and 3 (91, 92), the IR (93, 94), VEGFR (95, 96), and IGF-1R (19, 20, 52–54, 97).

Translocation of the IGF 1R to the nucleus in cancer cells is induced by IGF-1 (20, 53, 98). Nuclear IGF-1R is more pronounced in cancer cell lines, including breast cancer, prostate cancer and sarcoma cells, compared to non-transformed cells (97). Furthermore, nuclear IGF-1R has been linked to a poor outcome for cancer patients and suggested to promote a more advanced disease stage (20, 53, 98, 99). Nuclear IGF-1R traffics from the plasma membrane (97) and the levels of IGF-1R nuclear translocation are proportional to ligand-induced kinase activation, because its translocation in cancer cells can be inhibited by xentuzumab, an IGF-1/2 neutralizing antibody, or by inhibition of IGF-1R endocytosis (20, 53, 54).

The precise mechanisms of IGF-1R import into the nucleus of normal and cancer cells are still unclear because the IGF-1R does not have a nuclear localization sequence (NLS) (53, 54) (**Figure 2B**). SUMOylation of the IGF-1R induced by IGF-1R internalization was proposed to be important (54), and IGF-1R translocation in cancer cells is facilitated by a specific subunit of dynactin p150Glued (52) (**Figure 2B**). The latter study showed that IGF-1-bound and internalized IGF-1R is transported within early endosome antigen 1 (EEA1)-positive vesicles (**Figure 2B**), it becomes positioned in the nuclear pore complex by β-importin, and is subsequently SUMOylated by RanBP2 for translocation into the nucleus (52). Suppression of any of the proteins involved in this import, leads to a significant decrease in nuclear IGF-1R. However, mutation of the SUMOylation lysine sites on IGF-1R did not abolish accumulation of IGF-1R in the nucleus (54), suggesting that additional import mechanisms exist. IGF-1R association with other proteins containing an NLS, such as IRS-1, which was previously shown to translocate to the nucleus in response to IGF-1, could also promote the import (100). It has also been suggested that heterodimerization with the IR, which occurs rapidly in response to Insulin stimulation (93) could promote nuclear import (55).

Nuclear IGF-1R may associate with DNA to enhance transcription (19, 54–56, 101), for example, by mediating the recruitment of RNAPol2 (20). Nuclear IGF-1R autoregulates its own expression in breast cancer cells depending on their estrogen receptor (ER) status (102) and binds the LEF1 transcription factor, which subsequently leads to upregulated cyclinD1 and axin 2 and cell proliferation (56). In HeLa cells, nuclear IGF-1R can increase the expression of SNAI2 (55), which is involved in EMT by suppressing E-cadherin expression (103). In prostate cancer cells nuclear IGF-1R facilitates expression of JUN and FAM 21, which are linked to cell survival, anchorage independent growth and cell migration, all of which are associated with advanced cancer stage (20). Nuclear IGF-1R is associated with proliferation of alveolar rhabdomyosarcoma cells (104) and contributes to chemoresistance in sarcomas and hepatocellular carcinoma (105, 106).

Overall, the results from recent studies suggest that nuclear IGF-1R facilitates an aggressive cancer phenotype. However, Aleksic et al. suggest that the sites of IGF-1R binding in DNA, and therefore the genes influenced by nuclear IGF-1R, might be cell type specific and that this could be defined by nuclear structure and chromatin organization (20). This is supported by the result of Sarfstein et al., which suggests that in presence of ER, nuclear IGF-1R cannot enhance its own expression (102).

## Going the Distance-Insulin-Like Growth Factor 1 Receptor Signals to the Mitochondria

While IGF-1 signaling in metabolism has been well studied (107), its contributions to mitochondrial function, maintenance and turnover is an emerging topic. Mitochondrial metabolism and oxidative phosphorylation (OXPHOS) provide building blocks and energy for all cellular functions (108). At the same time, reactive oxygen species (ROS), which are a normal by-product of OXPHOS are neutralized to avoid accumulation and cell damage (109). Mitochondria synthesis (mitochondrial biogenesis) and the regulation of numbers and quality (mitophagy linked to mitochondrial fission and fusion) are well-orchestrated processes. The importance of mitochondrial quality control and mitochondrial homeostasis in the maintenance of healthy tissues is well documented (108, 110). Impaired mitophagy can lead to the accumulation of dysfunctional mitochondria and oxidative stress, which is associated with various diseases including neurodegeneration, diabetes, heart disease and cancer (108, 111–115).

IGF-1 signaling and a functional IGF-1R is essential for mitochondrial biogenesis through inducing the transcriptional mediators Peroxisome proliferator-activated receptor gamma coactivator 1 β (PGC1β) and PGC-1-related coactivator (PRC) (116, 117) (**Figure 2C**). Suppression of the IGF-1Ror the PI3-Kpathway using the IGF-1R kinase inhibitor BMS-754807 or LY294002, respectively, leads to a reduction in mitochondrial mass and biogenesis (116). IGF-1 also induces the mitophagy receptor BNIP-3 (116) through GSK-3β mediated activation of NFE2L2/Nrf2 (118) (**Figure 2C**). This highly conserved signaling pathway is conserved from *C. elegans* where it coordinates mitochondrial biogenesis with mitophagy and thereby controls cellular metabolism that is ultimately linked with lifespan (119, 120). In mammalian cells (normal or transformed), IGF-1-mediated regulation of mitochondrial biogenesis and mitophagy is more complex that in *C. elegans*. In metazoans, it needs to be integrated with metabolic status and IGF-1-stimulated mTORC1 actions in suppressing cellular macro-autophagy (121, 122). Although IGF-1 signaling may be critical for both mitochondrial biogenesis and basal mitophagy, it is not however easy to distinguish specific signals for mitophagy from general autophagy. Moreover, IGF-1 signals may control basal mitochondria health and the triggering of mitophagy in very specific cellular contexts such as cell division or differentiation.

Further evidence for an essential IGF-1 signal in maintaining healthy mitochondria comes from the IGF-1-inducible mitochondrial UTP importer, pyrimidine nucleotide carrier 1 (SLC25A33/PNC1) that is required for maintaining mitochondrial RNA and DNA (123, 124) (**Figure 2A**). Suppression of PNC-1 results in cellular accumulation of ROS under normal oxygen conditions, an increase in glycolysis and a profound induction of EMT in cancer cells (124).

Overall, it will be important to establish how IGF-1 signals and IGF-1R activity support mitochondrial function in normal cells and in phenotypically distinct cancer cells, and whether an essential component of these signals is to maintain a healthy pool of mitochondria that would prevent cancer aggressiveness that is associated with hypoxia, mitochondria dysfunction and an accumulation of cellular ROS.

### Where to go From Here?—Remaining Questions in the Field

This review summarizes current knowledge on IGF-1R trafficking and signaling to and from intracellular compartments. Overall, the potential for intracellular IGF-1R signaling adds complexity to understanding and modulating IGF-1 actions in physiological and patho-physiological conditions. For example, efforts to inhibit IGF-1R signaling at the plasma membrane are not very effective, as is evident from the poor success of mAb in targeting the IGF-1R in cancer. One explanation for this is that continued signaling from intracellular pools of IGF-1R in association with specific organelles or protein signaling complexes may circumvent plasma membrane targeting. Correlating IGF-1R location and activity at the Golgi or in the nucleus with a specific subset of cancer may be a valuable biomarker for targeting IGF-1R in cancer (125). Therefore, if IGF-1R trafficking to and signaling from intracellular compartments determines its activity in cancer and contributes to an aggressive cancer behavior (20, 22), it is now important to identify the molecular regulators of IGF-1R trafficking. The functions of these proteins in selecting incoming receptors and regulating their cellular distribution and localization may the key to cellular signaling responses. Illuminating the mechanisms of IGF-1R trafficking and endosomal sorting would provide new insights on IGF signaling in normal cells and cancer cells, and may also identify potential co-targets for pharmacological intervention in cancer. Targeted therapy against proteins facilitating IGF-1R location and activity in the Golgi or the nucleus, or enhancing IGF-1R sorting toward proteosomal degradation may be beneficial in certain subtypes of cancer. Moreover, the presence of the IGF-1R at the Golgi may have potential to identify cancer subtypes where membrane targeting would not be effective. Our data on IGF-1R derived Golgi signaling also suggest that removing the receptor is important to suppress IGF-1 signaling. However, it is not yet clear whether specific antibodies that promote IGF-1R internalization could be used to direct it to the degradation machinery. It may be necessary to identify the key regulators of receptor trafficking to achieve selectivity here. 125.

## Author Contributions

LR and RO’C contributed to the writing of the article. All authors contributed to the article and approved the submitted version.

## Conflict of Interest

The authors declare that the research was conducted in the absence of any commercial or financial relationships that could be construed as a potential conflict of interest.
